# The distracted mind on the wheel: Overall propensity to mind wandering is associated with road crash responsibility

**DOI:** 10.1371/journal.pone.0181327

**Published:** 2017-08-03

**Authors:** Cédric Gil-Jardiné, Mélanie Née, Emmanuel Lagarde, Jonathan Schooler, Benjamin Contrand, Ludivine Orriols, Cédric Galera

**Affiliations:** 1 University Hospital of Bordeaux, Pellegrin Hospital, Emergency Department, Bordeaux, France; 2 INSERM, ISPED, Centre INSERM U1219-Epidemiologie-Biostatistique, Equipe Prévention et prise en charge des traumatismes, Bordeaux, France; 3 Department of Psychological & Brain Sciences, University of California, Santa Barbara, California, United States of America; 4 University of Bordeaux, Charles Perrens Hospital, Department of Child and Adolescent Psychiatry, Bordeaux, France; Beihang University, CHINA

## Abstract

The role of distractions on attentional lapses that place road users in higher risk of crash remains poorly understood. We aimed to assess the respective impact of (i) mind wandering trait (propensity to mind wander in the everyday life as measured with a set of 4 questions on the proportion of time spent mind wandering in 4 different situations) and (ii) mind wandering state (disturbing thoughts just before the crash) on road crash risk using a comparison between responsible and non-responsible drivers. 954 drivers injured in a road crash were interviewed at the adult emergency department of the Bordeaux university hospital in France (2013–2015). Responsibility for the crash, mind wandering (trait/state), external distraction, alcohol use, psychotropic drug use, and sleep deprivation were evaluated. Based on questionnaire reports, 39% of respondents were classified with a mind wandering trait and 13% reported a disturbing thought just before the crash. While strongly correlated, mind wandering state and trait were independently associated with responsibility for a traffic crash (State: OR = 2.51, 95% CI: 1.64–3.83 and Trait: OR = 1.62, 95% CI: 1.22–2.16 respectively). Self-report of distracting thoughts therefore did not capture the entire risk associated with the propensity of the mind to wander, either because of under-reported thoughts and/or other deleterious mechanisms to be further explored.

## Introduction

Every year in the world, 1.2 million people lose their lives on roads [1]. In Western Countries comprehensive laws have been implemented to target classical crash risk factors such as alcohol consumption, seat-belts non-use, helmet wearing, speed or cellular use when driving. The introduction of these laws has greatly improved road safety, crash mortality and morbidity, with a thirty times reduction in mortality per kilometer travelled since the years 1950s [2]. Further progress in road safety is more and more difficult to achieve and needs to focus on new risk factors and attentional determinants are gaining scientific interest in this context [3]. Indeed, a defect of attention (i.e. inattention) could be involved in roughly half of car crashes [4]. Inattention at the wheel involves a large range of problems including distractions from external (e.g. phone cell, passengers, in-vehicle motor actions) and internal (e.g. tiredness, drowsiness, attention deficit hyperactivity disorder) sources. Recently a common form of internal distraction called mind wandering (i.e. a thought unrelated to the current task [5]) has been shown to be associated with a significant increase in crash risk in both epidemiological and experimental studies [6–10]. Mind wandering can be described both as a state, for example the fact one is distracted by disturbing thoughts at a given point in time or as a trait, which corresponds to variations in individuals’ chronic tendency to be engaged in mind wandering. To our knowledge, we previously conducted the sole epidemiological study that has assessed in real life conditions the impact of self-reported mind wandering state just before accidents [5]. We found a positive relationship between disturbing thoughts and responsibility in the crash. If causal, the association between mind wandering and road crash opens a new avenue for interventions of several kind, from advanced driver assistance systems to psychological training aimed to improve thought control. Our results could however be explained by an information bias (i.e. desirability bias), responsible drivers being more likely to report disturbing thoughts following a self-explaining process. Showing an inter-individual heterogeneity towards mind wandering and its impact on crash risk would provide further evidence in favor of a causal link. We therefore replicated and extended our study (i.e. a case-control responsibility study in a sample of injured drivers) with the objective of investigating the association between mind wandering, both as a state and as a trait, and the risk of being responsible for a motor vehicle crash.

## Methods

### Study design and settings

We conducted a responsibility case-control study in an adult emergency department (ED) of Bordeaux university hospital, which serves both rural and urban areas counting 1.4 million inhabitants. The study was conducted from March 2013 to January 2015. Research assistants interviewed patients recently admitted for a crash. We compared the frequency of exposures (Mind wandering variables and confounders) between drivers responsible for the crash (cases) and not responsible for the crash (controls).

### Participants

All patient admitted in the ED in the first 24 hours after a road traffic accident were eligible for study inclusion if they were drivers legally of age (i.e. ≥ 18 years in France) and able to answer the interviewer (speaking French and with a Glasgow coma score of 15 when interviewed). A total of 1103 patients were assessed for eligibility. Among them 12 were ineligible and 11 refused to participate. 126 patients were secondarily excluded from the analysis because of incomplete data needed to score their responsibility. Finally, the sample for analysis comprised 954 patients.

### Outcome variable

#### Responsibility for the crash

We determined responsibility levels in the crash using a standardized method adapted from the Robertson and Drummer crash responsibility tool [11]. The adapted method takes into account mitigating factors likely to reduce driver responsibility: road environment, vehicle related factors, traffic conditions, type of accident, traffic rule obedience, and difficulty of the driving task. Each factor scores from 1 (not mitigating, i.e. favorable to driving) to 3 or 4 (mitigating, i.e. not favorable to driving). All six scores are summed to provide a responsibility score (multiplied by 8/6 to be comparable with the eight factor score proposed by Robertson and Drummer). This method has been previously validated in the French context [5,12–15]. Indeed, two factors such as “level of fatigue” and “witness observation” are unavailable in French Police records. The higher the score, the lower the responsibility. Responsibility scores are classified into three categories: 8–12 = responsible; 13–15 = contributory; >15 = not responsible. Drivers displaying any degree of responsibility for the crash were classified as cases (score ≤15); drivers who were judged not responsible (score >15) served as controls. The interviewer was unaware of the responsibility status while interviewing the participants since responsibility scores were computed during the analysis.

### Risk factors

Participants were asked to describe their thoughts just before the crash and the question was coupled with a numeric scale from 0 to 10 that captured the self-estimated level of perturbation. In order to reduce memory bias and halo effect, two opportunities were offered during the interview to report thoughts which were subsequently classified as being related or not to driving. The Mind Wandering State was defined as the report of any thought unrelated to driving. A *Disturbing Thought (DT)* corresponded to a Mind Wandering State with a perturbation rating higher than 4. Perturbation level was indeed the answer to “How disturbed / distracted was this thought?”. *Mind Wandering Trait* was built from a scale comprising four items selected based on their clinical significance. Two items are part of the Day Dreaming Frequency Scale (DDFS): *Daydreams and fantasies make up X % of the day*, and *Recalling things from the past*, *thinking of the future*, *or imagining unusual kinds of event occupies X% of my day* [16]. Two items were developed from literature data: *In general*, *when you drive*, *how often do you happen to think about something else*? And *In general*, *when you read*, *how often do you happen to think about something else*. For each question, the related time spent each day was measured from 0 to 100 percent. If the frequency was higher than 50% for at least one item, the patient was defined as in the high category of the boolean MWT variable.

The analysis also included well-known risk factors for road crash and potential confounders such as patient characteristics (age, sex, socioeconomic category), alcohol consumption during the 6 hours before the crash and self-reported psychotropic drug use the day before accident. Characteristics of the crash were also reported (location, vehicle type). The variable *Distractive Activity* was obtained by asking participants about their activities just before the crash (this included use of a mobile phone, listening to radio/television, talking with or listening to a passenger, manipulation of electronic devices, manipulation of objects, grooming, smoking, eating, drinking, reading). Patients were also asked to evaluate their pain at the time of the interview with a numeric scale; A painful participants was defined as with a self-rated pain value strictly superior to 3. Participants were also asked whether they had been distracted by a distracting event that occurred inside or outside the vehicle. Sleep Deprivation was evaluated with The Epworth Sleepiness Scale (ESS) [17].

### Statistical analysis

Univariate analysis was conducted to investigate the link between crash responsibility and risk factors using Student t-test for continuous variable and Chi-square test for categorical variable. Multivariate analysis was then performed with a step by step backwards selection procedure keeping all significant variables (p < 0.05) and all confounders (variation of β > 20%). We then tested interactions between independent variables kept in the final model. Finally, we performed sensitivity analyses to assess the robustness of the results: 1. by stratifying on pain; 2. by changing the cut-off for responsibility score to 14 and 16; 3. by stratifying on the existence of chronic disease. Data were analyzed using SAS Software (v9.4, SAS Institute inc).

### Ethical approval

Study protocol was approved by the French data protection authority and the regional ethics committee (Comité de protection des personnes Sud-Ouest Outre-Mer III). All participants gave written informed consent.

## Results

The Study included 954 patients. Table 1 describes the characteristics of the sample and the main factors included in the analysis. Briefly, participants were principally male (61%), aged on average 38 years (SD = 15) and 46% (n = 440) were classified as responsible.

**Table 1:** Characteristics of the study population—Comparison between drivers responsible and not responsible for the road crash (n = 954).

		Population	R (%)	NR (%)	p
Total		954	440	514	
**Socio-demographic characteristics**					
Sex (n = 952)	Woman	371 (39.0)	177 (40.2)	194 (37.7)	NS
Age (n = 938)					
Under 24 y		216 (22.6)	111 (25.2)	105 (20.4)	**< 10**^**−2**^
[25–44]		430 (45.1)	173 (39.3)	257 (50.0)	
[45–59]		213 (22.3)	115 (26.2)	98 (19.1)	
More than 60 y		95 (10.0)	41 (9.3)	54 (10.5)	
Professionnal activity (n = 951)					**<10**^**−2**^
No		148 (15.6)	86 (19.5)	62 (12.1)	
Yes		803 (84.4)	351 (80.5)	452 (87.9)	
Professional Driver		330 (34.9)	142 (32.3)	188 (36.5)	NS
Vehicule type					**< 10**^**−2**^
Bike		268 (28.1)	156 (35.5)	112 (21.8)	
Motorcycle / Scooter		325 (34.1)	148 (33.6)	177 (34.4)	
Car		315 (33.1)	118 (26.8)	197 (38.3)	
Commercial vehicle		45 (4.7)	18 (4.1)	27 (5.3)	
Pain (Numeric Scale > 3)		645 (67.6)	283 (64.3)	362 (70.4)	**< 0.05**
**External distractions**					
Verbal activity		267 (28.0)	113 (25.7)	154 (30.0)	NS
Movement while driving		31 (3.2)	14 (3.2)	17 (3.3)	NS
Any external distractor		283 (29.7)	119 (27.0)	164 (31.9)	NS
Listening to music		242 (25.4)	98 (22.3)	144 (28.0)	**< 0.05**
Distracting event outside the vehicle		292 (30,6)	126 (28.6)	166 (32.3)	NS
**Attention-related variables**					
Disturbing thoughts before the crash(n = 953)		129 (13.5)	88 (20.0)	41 (8.0)	**< 10**^**−2**^
Mind Wandering Trait (n = 930)		372 (39.0)	206 (40.8)	166 (32.3)	**< 10**^**−2**^
ADHD		76 (8.0)	41 (9.3)	35 (6.8)	NS
**Well-known Risk-factors**					
ESS score >8		383 (40.1)	151 (34.3)	124 (24.1)	**< 10**^**−2**^
Alcohol consumption in the last 6 hours		65 (6.8)	45 (10.2)	20 (3.9)	**< 10**^**−2**^
Psychotropic drug on the crash day		64 (5.4)	37 (8.4)	27 (5.3)	NS
**Time of crash**					NS
[20.00–7.59]		217 (22.8)	91 (20.7)	126 (24.5)	
[8.00–19.59]		736 (77.2)	348 (79.3)	388 (75.5)	

R: Responsible for the crash

NR: Not Responsible for the crash

ADHD: Attention Deficit Hyperactivity Disorder

ESS: Epworth Sleepiness Scale

NS: Non significant

Multivariate logistic regression (Table 2) showed that Disturbing Thoughts and Mind Wandering Trait were independently and strongly associated with responsibility (Adjusted odds-ratio [95% CI]: 2.51 [1.64–3.83] and 1.62 [1.22–2.16] respectively). Alcohol use within 6 hours before the crash, sleep deprivation assessed by Epworth Scale, vehicle type and age categorized in three groups were also associated with crash responsibility. We forced in the final model the sex of the patients and the existence of an external event since they are theoretically important variables. We found no significant interactions. Sensitivity analyses (stratified on pain on arrival to the ED, changing to cut-off for responsibility score or chronic disease) showed the same pattern of results (Table 3).

**Table 2:** Multivariate analysis of factors associated with crash responsibility (n = 926).

		Responsible for the crash N (%)	Crude OR	95% CI	Adjusted OR*	95% CI
**Disturbing Thoughts**		88 (68.2)	2.68	[1.75–4.10]	2.51	[1.64–3.83]
**Mind Wandering Trait**		206 (55.4)	2.00	[1.50–2.66]	1.62	[1.22–2.16]
**Epworth Sleepiness Scale score > 8**		151 (39.4)	1.65	[1.24–2.20]	1.40	[1.03–1.91]
**Alcohol consumption in the last 6 hours**		55 (69.2)	2.33	[1.34–4.08]	2.68	[1.49–4.84]
**Vehicle type**	Car	118 (37.4)	Ref.		Ref.	
Commercial vehicle		18 (40.0)	1.00	[0.49–2.03]	1.16	[0.57–2.34]
Bike		156 (58.2)	2.35	[1.63–3.38]	2.15	[1.50–3.08]
Motorcycle / scooter		148 (45.5)	1.41	[1.00–2.00]	1.45	[1.02–2.07]
**Age**	Under 24 y	111 (51.4)	1.57	[1.13–2.18]	1.44	[1.01–2.04]
25 y– 44 y		173 (40.2)	Ref.		Ref.	
45 y– 59 y		115 (54.0)	1.60	[1.25–2.42]	1.94	[1.35–2.77]
More than 60 y		41 (43.2)	1.13	[0.72–1.77]	1.09	[1.05–2.10]
**Time of Accident**	[20.00–7.59]	91 (41.9)	Ref.		Ref.	
[8.00–19.59]		348 (47.3)	1.24	[0.91–1.69]	1.49	[1.05–2.10]

*Odds-ratio for responsibility in road accidents adjusted on: sex, age, disturbing thoughts, mind wandering, Epworth scale, last Alcohol consumption < 6h, vehicle type, external event, time of accident.

**Table 3:** Sensitivity analyses of responsibility risk factors in road accidents.

	OR*	IC à 95%
***Pain patients (n = 614)***		
Disturbing Thoughts	2.34	[1.40–3.90]
Mind Wandering trait	1.45	[1.01–2.07]
***Painless patients (n = 297)***		
Disturbing Thoughts	2.73	[1.24–6.03]
Mind Wandering trait	2.14	[1.28–3.58]
***Responsibility score limit at 14 (n = 911)***		
Disturbing Thoughts	2.16	[1.44–3.24]
Mind Wandering trait	1.55	[1.14–2.12]
***Responsibility score limit at 16 (n = 911)***		
Disturbing Thoughts	2.43	[1.53–3.86]
Mind Wandering trait	1.53	[1.14–2.05]
***Patients with Chronic Disease (n = 392)***		
Disturbing Thoughts	2.72	[1.32–5.60]
Mind Wandering trait	1.79	[1.15–2.79]
***Patients without chronic disease (n = 519)***		
Disturbing Thoughts	2.48	[1.46–4.20]
Mind Wandering trait	1.53	[1.03–2.26]

*Odds-ratio for responsibility in road accidents adjusted on: sex, age, disturbing thoughts, mind wandering, Epworth Sleepiness Scale, last Alcohol consumption < 6h, vehicle type, external event.

## Discussion

Both current mind wandering before the crash (reported as disturbing thoughts) and the general propensity to mind wander in the everyday life were independently and strongly associated with responsibility for the crash. This result remained significant even when adjusting for a large range of potential confounders including classic risk factors such as recent alcohol consumption and sleepiness.

One strength of the study is the responsibility case-control design that avoids including participants not involved in any crash and who therefore may differ for the population of interest (drivers driving at the time of the study). Using this methodology with, for the first time to our knowledge, a concomitant assessment of mind wandering trait and state is another important step. In a previous study, we evidenced an association between crash responsibility and self-reported disturbing thoughts before the crash [5] but could not exclude a potential desirability bias. Participants might have reported these thoughts as a reason for exonerating or minimizing their responsibility. The further association with mind wandering trait strengthens the plausibility of a causal association between mind wandering and responsibility for the crash.

Among limitations, the study may suffer from a selection bias as participants are exclusively recruited in the emergency ward, missing minor accidents. Severely injured patients were also not included in the study because of their inability to provide consent and answer our questionnaire.

The retrospective and self-reported data collection could misestimate the prevalence of disturbing thoughts because of a recall or desirability bias. A memory bias could also distort drivers’ reports of the sequence of events because of short duration between the crash and the period in which we collected the thoughts. However since there is little way to cope with these phenomenon in ecological circumstances we think that self-reporting remains so far the best way to evaluate mind wandering state in this population.

In addition to being the independent association of mind wandering trait and state, we found that individuals with who scored high for mind wandering trait were more likely to report disturbing thoughts. Although correlational, this confluence of findings supports the same causal pathway: mind wandering may truly be an intrusive process that disrupts the automated driving task when intense.

In the context of driving, task monotony may trigger the mind wandering process [18]. The subsequent competition between mind wandering and selective attention to the external world (i.e. the car environment including the road and its hazards) may then heighten the risk for crashes. The driver will indeed be less able to notice and integrate all the information needed to drive safely and in a responsive manner. As already hypothesized, mind wandering impacts the neurocognitive processing of external events, and specifically leads to perceptual decoupling [19] whereby attention to external stimuli is dampened [20]. Mind wandering may also lead to a relative disengagement in the driving task through failures in the management of the dynamics of attention and distraction. Finally, mind wandering while driving may also lead to difficulties in prioritizing actions and in switching from one task to another [21].

Only thoughts self-rated as “disturbing” were however associated with an increased probability of being responsible in the crash. Internally and externally directed cognition are indeed not necessarily incompatible and both can co-occur with no damaging interferences. It has been hypothesized that it is the case as long as no high levels of intentionality are involved [22]. It seems however that non-intentional intruding thoughts can also affect the crash risk.

Previous work on the risk of road crash for people with chronic conditions showed higher risks for driver with depression [23–25]. Such a result have been considered as counter intuitive given the low propensity of depressed driver to speed or to engage in risk-taking behaviours. While probably partly associated with the impact of anti-depressant drugs [25], this may be explained by the impact of internal thoughts. There is however a difference between our abilty to let our mind wander without ruminating and perseverative cognition with dysfunctional intrusive thoughts [26]. Such a distinction between cognitive wandering state were also suggested for patients with attention-deficit/hyperactivity disorder (ADHD) [27].

Because of its strong association with responsibility and the large proportion of driver concerned, mind wandering is likely to play a very significant role in road traffic crash burden. Safe driving may be heavily threatened by disturbing thoughts. Thus, sensitivity analyses presented in Table 3 underlines this important role of both Mind Wandering trait and Disturbing thoughts in the occurrence of a traffic accident. MW and DT remains significantly associated with responsibility after stratification on variables that could be potential confounders. This tends to confirm our hypothesis.

This underscores the necessity to explore ways to cope with mind wandering while driving. Interesting laboratory and field observations [28] suggested that there is an irresistible urge to let one’s thoughts drift and it would therefore be mistaken to thing that it is possible to avoid and control the focus of the driver at any point of the driving time.

Onboard screening and warning for hampering mind wandering may be one possible solution. Electronic devices (including eye trackers) able to detect physiological modifications (i.e. pupil diameter and eye movements) during mind wandering episodes may allow the development of effective assistance systems [29–34]. Second, lowering the occurrence of disruptive mind wandering while driving could also be beneficial [35,36]. Mindfulness and attention therapies in high mind wanderers [37] is promising and its potential value should be assessed both with epidemiological and driving simulators samples. Third, autonomous (self-driving) cars could represent a desirable evolution circumventing risky human factors including mind wandering and attention issues.

Interestingly, recent experimental studies have shown that mind wandering preferentially disrupts processing in the left visual field. Such results may have implications for road safety [38]. First, researchers with detailed data on the crash event would be invited to assess any crash configuration asymmetry linked with factors associated with Mind Wandering, for example age. This could also trigged further research on the differences between left-handed and right-hand-drive and left-hand-drive countries.

## Conclusions

Our study is the first to show the combined role of mind wandering trait and acute disturbing thoughts in the risk of road traffic crashes. The mind wandering phenomenon, through a potential attributable risk regarding car crashes around 10%, appears to be of utmost importance in the context of road safety. Engagement in the driving task may be heavily threatened by disturbing thoughts. This underscores the necessity to explore ways to cope with the phenomenon. Targeting current mind wandering onboard may be a first possibility. Second, preventing the occurrence of inadequate mind wandering while driving could be beneficial. Mindfulness and attention therapies in high mind wanderers is promising and need to be experimented whether in epidemiological or in driving simulators samples. Beyond road safety, the mind wandering phenomenon deserves consideration as a risk factor for accidents and trauma due to lowered attention to the task at hand.
